# Detection of *Clavibacter michiganensis* subsp. *michiganensis* in viable but nonculturable state from tomato seed using improved qPCR

**DOI:** 10.1371/journal.pone.0196525

**Published:** 2018-05-03

**Authors:** Sining Han, Na Jiang, Qingyang Lv, Yumin Kan, Jianjun Hao, Jianqiang Li, Laixin Luo

**Affiliations:** 1 Department of Plant Pathology and MOA Key Lab of Pest Monitoring and Green Management, College of Plant Protection, China Agricultural University, Beijing, P. R. China; 2 Beijing Key Laboratory of Seed Disease Testing and Control (BKL-SDTC), China Agricultural University, Beijing, P. R. China; 3 School of Food and Agriculture, The University of Maine, Orono, Maine, United States of America; Dong-A University, REPUBLIC OF KOREA

## Abstract

*Clavibacter michiganensis* subsp. *michiganensis* (*Cmm*) is a seed-borne pathogen that causes bacterial canker disease of tomato. *Cmm* is typically detected in tomato seeds using quantitative real-time polymerase chain reaction (qPCR) combined with culture-based isolation. The viable but nonculturable (VBNC) state of *Cmm* may result in the underestimation or false negative detection of the pathogen. In the present study, propidium monoazide (PMA) and its improved structure PMAxx were used to pretreat *Cmm* prior to DNA extraction, followed by qPCR. Both PMA and PMAxx could bind to the chromosomal DNA of dead bacterial cells and therefore block DNA amplification by PCR. This effect, however, does not occur in living bacterial cells, as the chemicals cannot penetrate through the undamaged cell membrane. Both viable and dead *Cmm* cells were treated with PMA and PMAxx at various concentrations. With this treatment, the range of the cell population was determined for effective detection. PMAxx showed a better discrimination effect than PMA on the viable and dead cells of *Cmm* and was therefore used throughout the present study. VBNC cells of *Cmm* (10^8^ CFU mL^-1^) was induced by 50 μM copper sulfate, which was detected at different sampling times up to a month by using both PMAxx-qPCR and flow cytometry assays. The optimal PMAxx concentration was 20 μM for detecting membrane-intact *Cmm* cells. High specificity and sensitivity were obtained at *Cmm* concentrations ranging from 10^3^ to 10^7^ CFU mL^-1^. The accurate and robust results of PMAxx-qPCR were confirmed by flow cytometry method to detect viable *Cmm* cells. Furthermore, the PMAxx-qPCR assay was successfully used in detecting VBNC *Cmm* cells in tomato seeds with as few as 10 seeds per set.

## Introduction

Bacterial wilt and canker of tomato (*Solanum lycopersicum*) is caused by the Gram-positive bacterium *Clavibacter michiganensis* subsp. *michiganensis* (*Cmm*) [1,2]. It is a devastating disease and has caused serious economic losses [3,4]. Disease symptoms include wilting of the whole plant, cankers and necrosis on stems and petioles as well as the reduced quantity and quality of fruit yield [5,6]. *Cmm* is a typical seed-borne pathogen, which can remain in or on the seed and is spread over a long distance through seed transportation [4,7]. Therefore, the certification of pathogen-free seed is an effective strategy to prevent and manage the disease, and this strategy is highly dependent on a reliable detection method of seed assay for pathogens [8].

In detecting *Cmm*, a number of techniques that have been used, including immune fluorescence staining, enzyme-linked immunosorbent assay (ELISA), southern hybridization, direct PCR, immunomagnetic separation, and loop-mediated isothermal amplification (LAMP) [9–12]. PCR is considered a promising detection approach because of its high throughput, sensitivity, specificity and convenient operation. However, PCR per se does not distinguish the viability of bacterial cells. Bio-PCR improves the accuracy in which culturable bacteria can grow on selective agar media prior to PCR. This method is currently recommended for detecting *Cmm* in tomato seed samples [13]. The bio-PCR protocols have been developed by the International Seed Testing Association (ISTA), the European and Mediterranean Plant Protection Organization (EPPO) and other international organizations [4].

The challenge in detecting *Cmm* is that the bacterium enters a viable but nonculturable (VBNC) state under stress conditions [14]. VBNC is defined as metabolically active but without the ability to grow on conventional media [15]. This characteristic is shared by many species of nonsporulating bacteria [16]. Studies have suggested that the ability to enter a VBNC state may be a survival strategy for bacteria to retain a low level of metabolic activity and maintain their cellular structure and virulence in adversity, and resuscitate under favorable conditions [17–19]. If VBNC occurs in *Cmm* from a commercial seed lot, then it is likely that the bacterial population can be underestimated by a culture-based method. Thus, the above detection method should be modified.

The VBNC state of bacteria is determined by examining cell viability. This examination can be accomplished by using techniques such as the LIVE/DEAD *Bac*Light Bacterial Viability Kit, cyanoditolyl tetrazolium chloride (CTC) and direct viable count (DVC), which is based on membrane integrity, respiration and responsiveness to nutritional stimuli. The LIVE/DEAD *Bac*Light Bacterial Viability Kit has been widely used to distinguish live cells [16,20]. Jiang et al. used this chemical treating bacteria prior to measuring the *Cmm* population with the flow cytometry method to count viable *Cmm* cells, combined with agar plating assay to enumerate VBNC cells [14]. However, this method cannot be used to detect the VBNC *Cmm* cells from a real sample, such as seed extract, because the flow cytometry method cannot differentiate between *Cmm* and other bacterial cells.

Several chemicals, such as ethidium monoazide (EMA) and propidium monoazide (PMA), are able to permeate bacterial cells with damaged membranes and covalently bind to double-stranded DNA upon exposure to bright visible light. The bounded DNA cannot be amplified in PCR and results in the amplification of only viable cells [21,22]. A quantitative real-time PCR (qPCR) combined with EMA (EMA-qPCR) or PMA (PMA-qPCR) has been developed to detect and quantify VBNC cells of *Listeria monocytogenes* and *Campylobacter* spp. [23,24]. PMA has been demonstrated as more effective than EMA in many PCR-based molecular assays [25], and PMA-qPCR was the most appropriate method for counting viable cells compared to acridine orange direct counts (AODC), DVC, and qPCR [26]. EMA-qPCR has also been used to quantify viable *Cmm* cells from pure *Cmm* microcosm [27].

The PMA-qPCR method is widely applied in the detection of viable cells in food-borne and environmental bacteria [26,28–32], while the application in plant pathogenic bacteria is less documented, particularly in plant seed health test. Both *Taq*Man real-time PCR assay and LAMP dilution endpoint assay combined with PMA have been used to detect the viable cells of *Xanthomonas hortorum* pv. *carotae* in carrot seeds [33] and *Acidovorax citrulli* in watermelon seeds [34]. However, no other studies have been reported by using the PMA-qPCR method to detect VBNC plant pathogenic bacterial cells. PMAxx, developed by Biotium in 2015, is an improved structure of PMA and can be used for viability PCR. It functions in a manner similar to PMA but has much greater activity and ability to distinguish between live and dead bacteria [35]. The objectives of the present study are to develop a protocol by using PMA-qPCR or PMAxx-qPCR, combined with a culture-based approach, for detecting *Cmm* cells in a VBNC state and apply the method for a tomato seed test.

## Materials and methods

### Ethics statement

The present study was conducted at Seed Health Centre of China Agricultural University (SHC-CAU). The microorganism used in the present study was isolated from diseased plants, and no endangered or protected species were involved.

### Bacterial strains and growing conditions

The *Clavibacter michiganensis* subsp. *michiganensis* strain BT0505 was isolated from an infected tomato plant in Inner Mongolia, China [27]. Bacterial cells were initially stored in 20% (v/v) glycerol at -80°C and streaked onto Luria-Bertani (LB) agar [27] at 28°C for 72 h. A single colony was selected to inoculate in 10 mL liquid LB and shaken at 120 rpm at 28°C for 24 h (log phase). Prior to use, the fresh live cells were washed three times with a 0.85% (w/v) NaCl solution, followed by centrifugation at 10000 ×g for 3 min. To assess the number of culturable cells, the cell suspension was tenfold diluted serially with sterilized distilled water. One hundred microliters of each diluted suspension was evenly spread onto a LB agar plate with three replicates. After incubation at 28°C for 72 h, plates with colony populations ranging from 30 to 300 were selected for bacterial enumeration.

### Preparation and counting of VBNC and dead cells of *Cmm*

*Cmm* cells at log phase (10^8^ CFU mL^-1^) were treated with 50 μM copper sulfate (CuSO_4_) at 28°C for 20 h to induce a VBNC state [14]. To detect VBNC cells, the induced microcosm was concentrated 100 times by centrifugation, subsequently plated onto LB agar to estimate the number of culturable cells, and analyzed with a flow cytometer to determine cell viability using the FACSCalibur system (BD Biosciences, San Jose, California, USA) according to a published protocol [14]. To obtain a VBNC *Cmm* suspension at 10^8^ CFU mL^-1^, the supernatant of copper-induced microcosm was collected, centrifuged and adjusted to OD_580_ = 0.5 with a sterile 0.85% (w/v) NaCl solution. The LIVE/DEAD *Bac*Light Bacterial Viability kit (Invitrogen, Carlsbad, California, USA) was used for bacterial staining prior to flow cytometry measurement [14]. *Cmm* cells treated with copper sulfate for 0, 3, 24, 72, 144, 240, 360, 480 and 720 h were measured with a flow cytometer for viability and on LB plates for culturability, respectively. If no colonies were observed on LB plates, then all viable cells determined by a flow cytometry were in a VBNC state.

To prepare dead bacterial cells, *Cmm* at log phase (10^8^ CFU mL^-1^) was transferred to Eppendorf tubes (1.5 mL) and heated at 80°C for 20 min in a dry bath. All dead cells were confirmed with a flow cytometer and dilution plating as described above. All the experiments were performed three times with two biological replicates.

### Optimization of photoactivatable dye for *Cmm* detection

One milligram of PMA (Biotium, Hayward, California, USA) was dissolved in 98 μL of sterile water with 20% dimethyl sulfoxide (DMSO) to obtain a 20 mM solution, and 20 mM PMAxx (a new and improved chemical of PMA) was purchased from Biotium Co., Ltd. Both dyes were stored in the dark at -20°C. To optimize the rate of photoactivatable dye for treating *Cmm* cells in quantitative real-time PCR, 1 mL of either culturable *Cmm* cells at the exponential phase or heat-killed cells (10^7^ CFU mL^-1^) was treated with PMA and PMAxx at a final concentration of 0, 2, 5, 10, 20, 30, 40 and 50 μM in Eppendorf tubes in the dark at room temperature (20 to 25°C) for 8 min. The tubes were placed in an ice bath with lids removed and exposed to the light of a halogen bulb (300 W) for 10 min at a distance of 20 cm to ensure the DNA free dye to be photolyzed. To ensure homogeneous exposure to light, the samples were shaken every 3 min during the light treatment. After photoactivatable dye treatments, the cells were collected for DNA extraction and purification using the E.Z.N.A. Bacterial DNA Kit (Omega Bio-tek, Norcross, Georgia, USA) according to the manufacturer's instructions. The purified DNA was resuspended in 50 μL DNA elution buffer and stored at -20°C for subsequent use.

In a PCR assay, *Cmm*-specific primers Spm4f/Spm2r were used, which targets the internal transcribed spacer (ITS) of ribosomal DNA [27]. The reaction for qPCR contained 2 μL of template DNA, 10 μL of 2 × SYBR Premix Ex Taq, 0.4 μL of 50 × ROX Reference Dye II (TaKaRa, Kusatsu, Shiga, Japan), 0.4 μM of each forward and reverse primer, and ultrapure water to bring up the final reaction volume to 20 μL. The qPCR was performed by using the ABI 7500 Fast fluorescence system (Applied Biosystems, Carlsbad, California, USA), and the following program setting: 1 min at 95°C; 40 cycles of 10 s at 95°C, 30 s at 60°C and 34 s at 72°C. All qPCR assays were performed three times.

To select the optimized concentrations of PMA and PMAxx for *Cmm* treatment, the quantification evaluation was calculated by dCt and ddCt values according to Randazzo [35]. Briefly, the dCt value was calculated by subtracting the Ct value of no-dye-treated samples from Ct values of dye-treated samples, while the ddCt value was calculated by subtracting the dCt of viable cells from the dCt of dead cells.

### Determination of viable *Cmm* cells in pure culture using PMAxx-qPCR

Viable cells of *Cmm* were measured by qPCR. *Cmm* cells at log phase were prepared as two groups: culturable cells (not treated) and dead cells (heat-killed), which were diluted by tenfold serial dilution to obtain a range from 10^8^ to 10^2^ CFU mL^-1^. The culturable and heat-killed cells were either treated with 20 μM PMAxx or without treatment for testing the influence of DNA amplification and the detection limit for PMAxx treatment prior to DNA extraction and qPCR assay. The number of culturable bacteria at each concentration was counted by dilution plating. A linear regression of standard curve was established between colony counts on plates and Ct values of PMAxx-qPCR.

To determine the effectiveness of the PMAxx-qPCR method in detecting VBNC *Cmm* cells, bacterial cells in log phase induced by 50 μM CuSO_4_ up to 30 days was used as the sample for analysis by PMAxx-qPCR and flow cytometry method. *Cmm* cells treated with copper for 0, 3, 24, 72, 144, 240, 360, 480 and 720 h were collected and used for plate count and PMAxx-qPCR as described above. The culturable *Cmm* cells were calculated by counting colonies on LB plates, and the viable cells were counted from the Ct value of qPCR. The number of VBNC cells was calculated by subtracting the number of culturable cells from the number of viable cells. Each assay was performed three times, and the experiments were conducted with two biological replicates.

### Detection of VBNC *Cmm* from tomato seed using PMAxx-qPCR

Culturable, VBNC and dead *Cmm* cells were prepared and verified as described above. *Solanum lycopersicum* ‘908’ (Changzhong Corp., Shanghai, China) was assayed by incubation on semi-selective medium mSCM [36], and quantitative real-time PCR to confirm the seeds as *Cmm* free. These seeds were artificially inoculated with culturable, VBNC and heat-killed *Cmm* cells (10^8^ CFU mL^-1^, OD_580_ = 0.5) for different treatments. The tomato seeds were inoculated artificially by vacuum processing [37,38]. An aspirator connected to a vacuum box was used to inoculate the *Cmm* cells on or in tomato seeds and vacuumed at -100 kpa for 5 min using SHB-III-type multi-use of recycled water vacuum pump (Great wall scientific industry, Zhengzhou, Henan, China). The inoculated seed was collected and placed on sterilized filter paper to air dry. Ten inoculated seeds from each treatment were randomly selected and ground by using a ball mill instrument (Retsch, Haan, Germany) in an Eppendorf tube. Five hundred microliters of 0.85% (w/v) NaCl solution was added to the broken seeds and incubated at room temperature (20 to 25°C) for 4 h for bacterial extraction. The seed extract was diluted 20 times with 0.85% (w/v) NaCl solution prior to PMAxx treatment. One milliliter of diluted seed extract was used for PMAxx pretreatment, DNA extraction, and qPCR as described above. Each assay was performed three times.

### Statistical analysis

Data analysis was performed by using the SPSS statistical program (Version 17.0, International Business Machines Corp., Armonk, New York, USA). The mean values were compared using Student’s *t-*test at a significance level α = 0.05.

## Results

### Optimization of photoactivatable dye for *Cmm* detection

The Ct values of viable cells treated with either PMA or PMAxx showed no significant difference compared with the control when PMA was less than 10 μM or PMAxx less than 20 μM. At 20 μM PMA, the Ct value of the viable cells was significantly higher than that with 20 μM PMAxx (Table 1).

**Table 1:** Cycle threshold (Ct) of quantitative real-time polymerase chain reaction for the detection of *Clavibacter michiganensis* subsp. *michiganensis* (10^7^ CFU mL^-1^) treated with propidium monoazide (PMA) and PMAxx (an improved PMA).

Photoactivatable dye	Concentration (μM)	Viable cells	Dead cells	ddCt^z^
		Ct^x^	Ct^y^	
PMA/ PMAxx	0	17.04 ± 0.12 a	16.85 ± 0.02 a	—
PMA	2	17.05 ± 0.19 a	29.16 ± 0.05 b	12.30
	5	17.16 ± 0.18 a	31.58 ± 0.17 cd	14.61
	10	17.39 ± 0.06 ab	34.41± 0.27 ef	17.21
	20	17.93 ± 0.28 c	34.28 ± 0.28 ef	16.54
	30	18.53 ± 0.24 d	33.08± 0.18 de	14.74
	40	18.82 ± 0.25 e	34.34 ± 0.32 ef	15.71
	50	19.22 ± 0.19 f	35.96± 0.38 f	16.93
PMAxx	2	17.29 ± 0.13 a	29.87 ± 0.14 bc	12.77
	5	17.47 ± 0.11 ab	31.14± 0.27 bcd	13.86
	10	17.35 ± 0.09 ab	33.98 ± 0.14 ef	16.82
	20	17.42 ± 0.01 ab	35.37 ± 0.24 ef	18.14
	30	17.66 ± 0.08 bc	34.64 ± 0.35 ef	17.17
	40	17.70 ± 0.38 bc	35.71± 0.32 ef	18.20
	50	18.00 ± 0.11 c	35.01± 0.44 ef	17.20

x and y = mean value ± standard deviation (SD).

^z^ddCt = dCt (Dead cells) dCt (Viable cells); dCt (Dead cells) = Ct (Dead cells with dye)–Ct (Dead cells without dye), dCt (Viable cells) = Ct (Viable cells with dye)–Ct (Viable cells without dye). Means followed by different letters are significantly different (*P* < 0.05)

For the heat-killed cells, the Ct value of *Cmm* treated with photoactivatable dye showed a significant increase compared to the control group. When the concentration of either PMA or PMAxx increased, the capacity of photoactivatable dye to eliminate the signal from dead cell DNA significantly increased. The Ct value of dead cells showed little change when the concentration of PMA and PMAxx was higher than 10 μM, and most values were greater than 34.00, except the values for dead cells treated with 30 μM PMA or 10 μM PMAxx (Table 1).

As the concentration of PMA increased, the ddCt value of PMA initially firstly increased and then decreased. The maximum value was calculated at 10 μM. After treatment with PMAxx at 40 μM, the ddCt value reached a maximum. However, this concentration of PMAxx was too high for viable cells. The second highest ddCt value was calculated when the PMAxx concentration was 20 μM (Table 1). This treatment did not affect viable cells but strictly inhibited dead cells (Ct value > 35). Based on the ddCt values calculated from viable and dead cells, the optimal concentrations of PMA and PMAxx were 10 and 20 μM. Because the ddCt value for 20 μM PMAxx treatment was higher than that for 10 μM PMA, it had no significant effect on the DNA amplification of viable *Cmm* cells. Thus, PMAxx was selected as the photoactivatable dye in the present study, and its optimal concentration was 20 μM.

### Determination of viable *Cmm* cells in pure culture using PMAxx-qPCR

**Fig 1:**
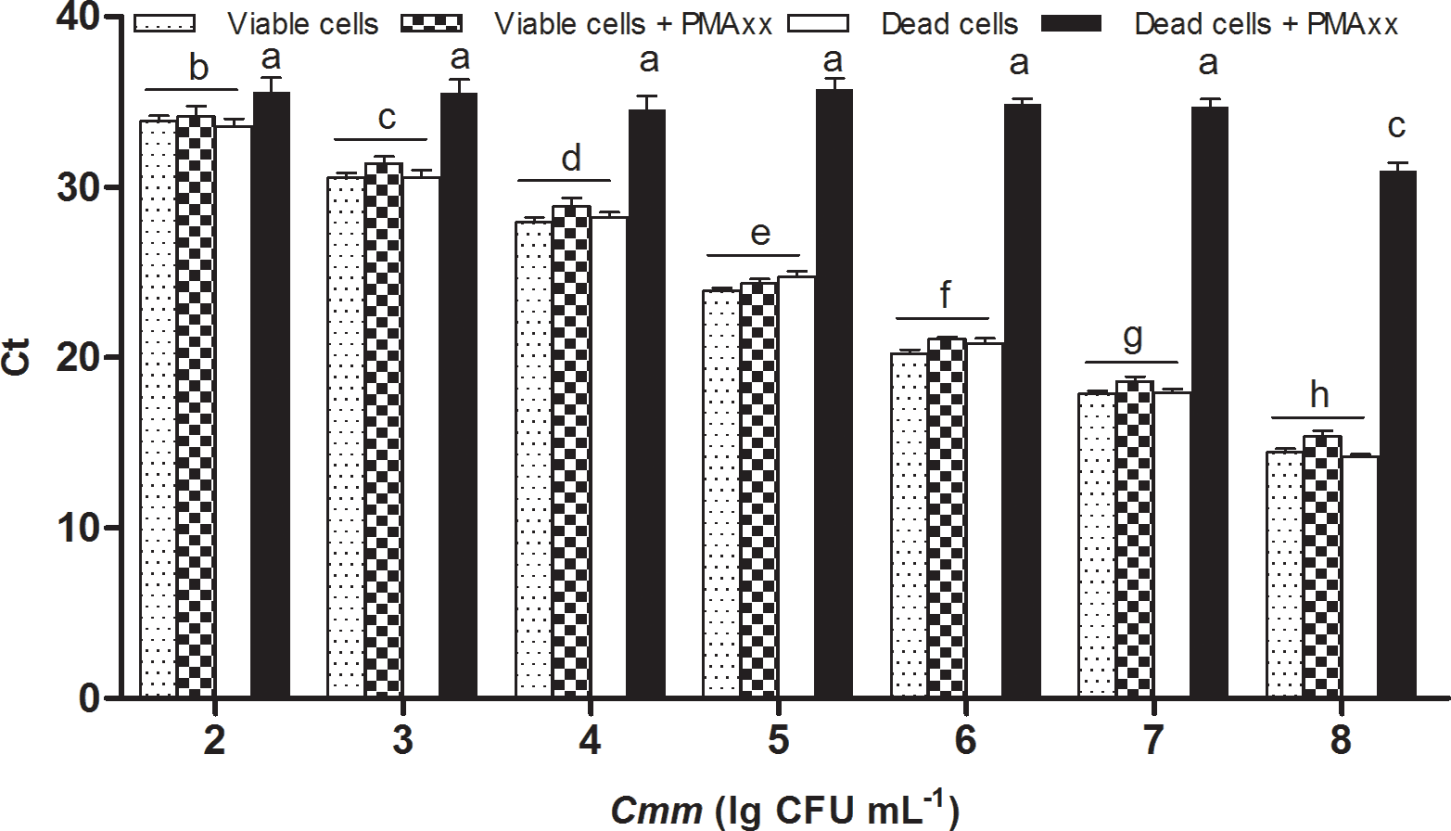
Specificity and sensitivity of PMAxx-qPCR assay in detecting viable cells of *Clavibacter michiganensis* subsp. *michiganensis* (*Cmm*). Viable or heat-killed *Cmm* cells at various concentrations were treated with 20 μM PMAxx, followed by DNA extraction and qPCR detection. Ct: threshold cycle of qPCR. CFU: colony forming unit. PMA: propidium monoazide. Columns and bars represent mean values and standard deviations. Means followed by different letters are significantly different (*P* < 0.05).

Tenfold serial dilutions of viable and dead *Cmm* cells treated with or without 20 μM PMAxx were used to evaluate the application range of the PMAxx-qPCR assay. The Ct values for PMAxx-treated viable cells at all concentrations were similar to those for untreated viable cells. For dead *Cmm* cells at 10^7^ CFU mL^-1^ or lower, the 20 μM of PMAxx was sufficient to inhibit the DNA amplification. However, when the concentration of heat-killed cells reached 10^8^ CFU mL^-1^, the Ct value significantly decreased compared to that at 10^7^ CFU mL^-1^ and lower concentrations (Fig 1). PMAxx at 20 μM did not bind to all the *Cmm* DNA from dead cells when the bacterial concentration was 10^8^ CFU mL^-1^.

**Fig 2:**
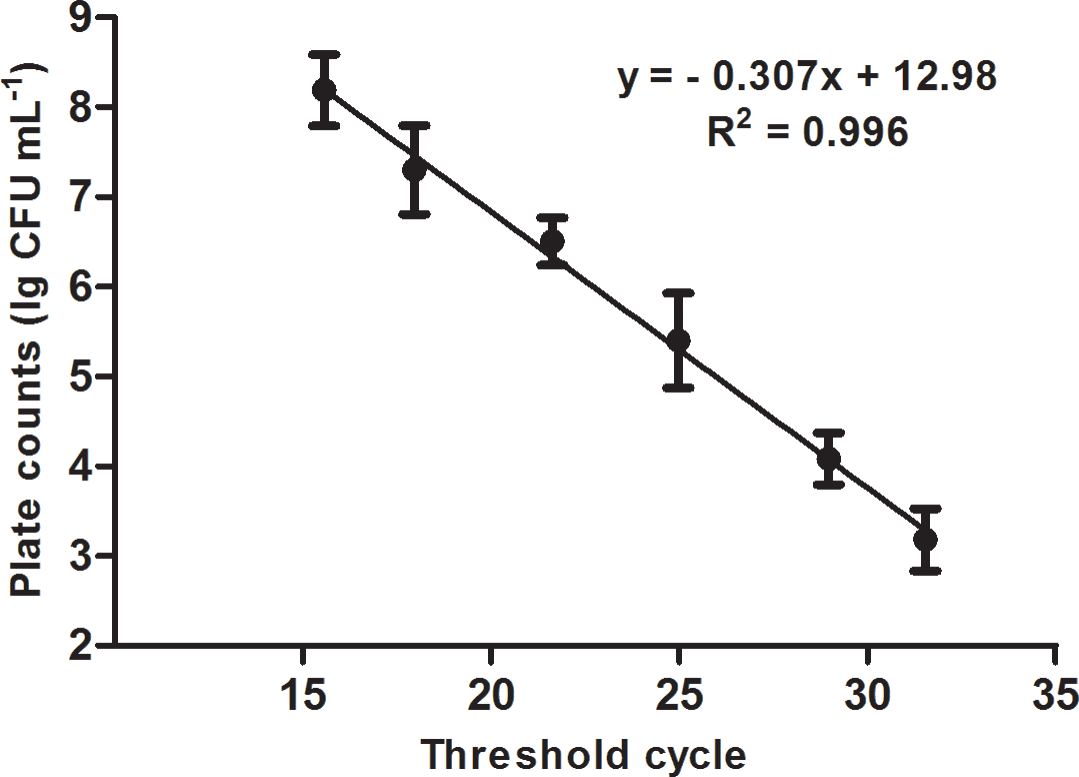
Correlation between bacterial population on agar plates and threshold cycle (Ct) of PMAxx-qPCR for culturable *Clavibacter michiganensis* subsp. *michiganensis* (*Cmm*). The final concentration of PMAxx used in bacterial cells treatment was 20 μM. The X-axis indicates the Ct value of PMAxx-qPCR and the Y-axis indicates the lg CFU mL^-1^ of bacterial cells.

There was a negative correlation between the number bacterial cells and Ct value of PMAxx-qPCR for culturable *Cmm* cells (Fig 2). Plotting the Ct value versus the log-concentration of *Cmm* yielded a straight-line regression, and the *R*^2^ was 0.996. Based on the standard curve, when the bacterial concentration ranged from 10^3^ to 10^7^ CFU mL^-1^, the number of viable *Cmm* cells could be calculated by using the Ct values obtained from PMAxx-qPCR.

### Comparison of flow cytometry and PMAxx-qPCR for the detection of VBNC cells

**Fig 3:**
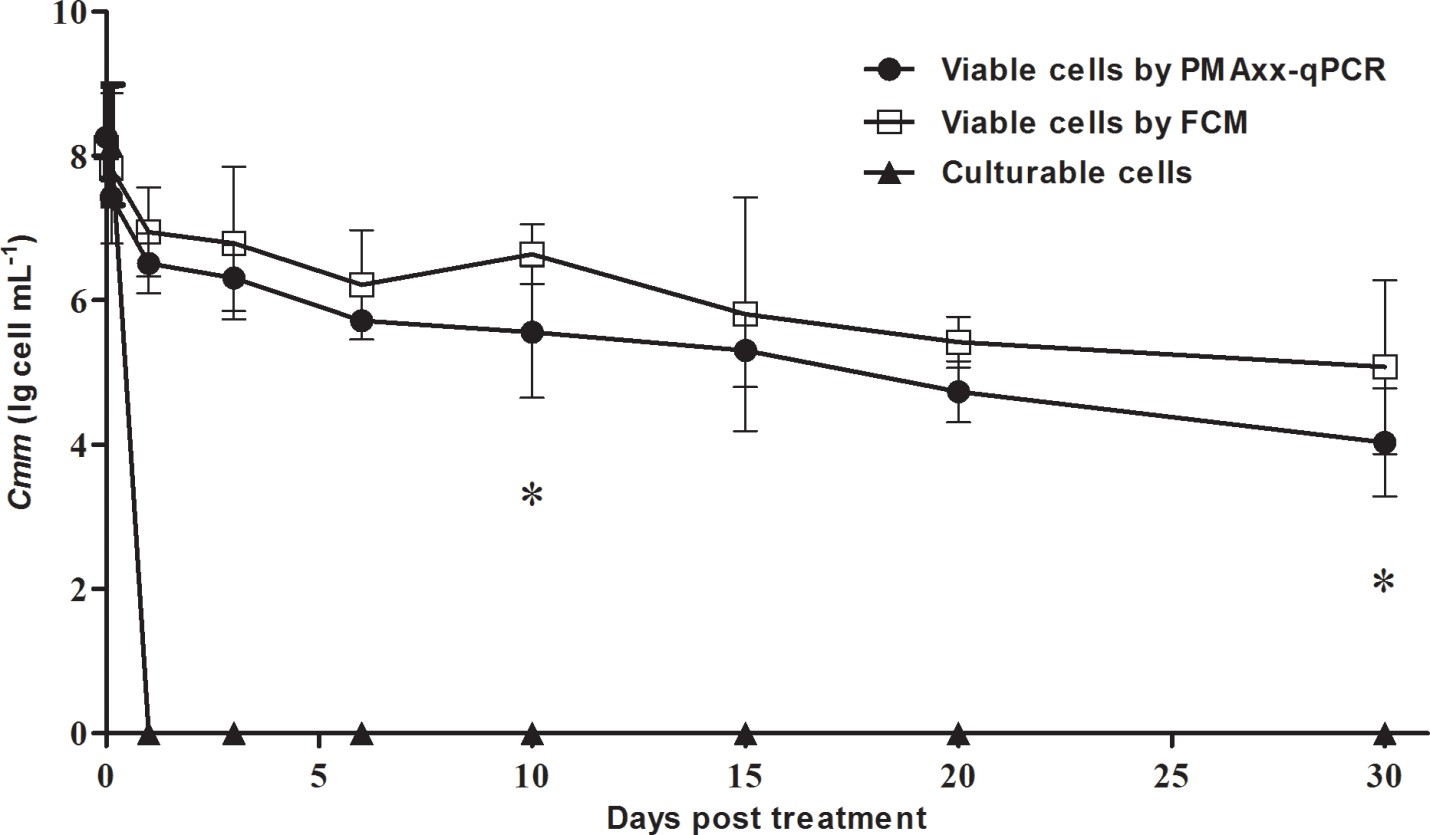
Detection of VBNC cells of *Clavibacter michiganensis* subsp. *michiganensis* (*Cmm*) by PMAxx-qPCR and flow cytometry (FCM). Viable and non-culturable (VBNC) cells of *Cmm* was induced by 50 μM CuSO_4_ in 0.85% NaCl. The bacterial cells were collected at 0 h, 3 h, 1 d, 3 d, 6 d, 10 d, 15 d, 20 d and 30 d, and used for viability detection by PMAxx-qPCR and flow cytometry method, while the culturability was determined on LB plates. Each data point represents the mean of two biological replicates. * indicates that the means were significantly different (*P* < 0.05) at the corresponding time point.

Both PMAxx-qPCR and flow cytometry methods exported a similar tendency of viable *Cmm* cells at each time point by detecting all viable cells, whereas the dilution plating method only showed the number of culturable cells. After 1-day incubation, no culturable cells were found on LB agar plates. In contrast, the viable cells detected by both PMAxx-qPCR and flow cytometry were above 10^4^ CFU mL^-1^ during 30 days of incubation, although this number decreased with time (Fig 3).

### Detection of VBNC *Cmm* from tomato seed using PMAxx-qPCR

**Fig 4:**
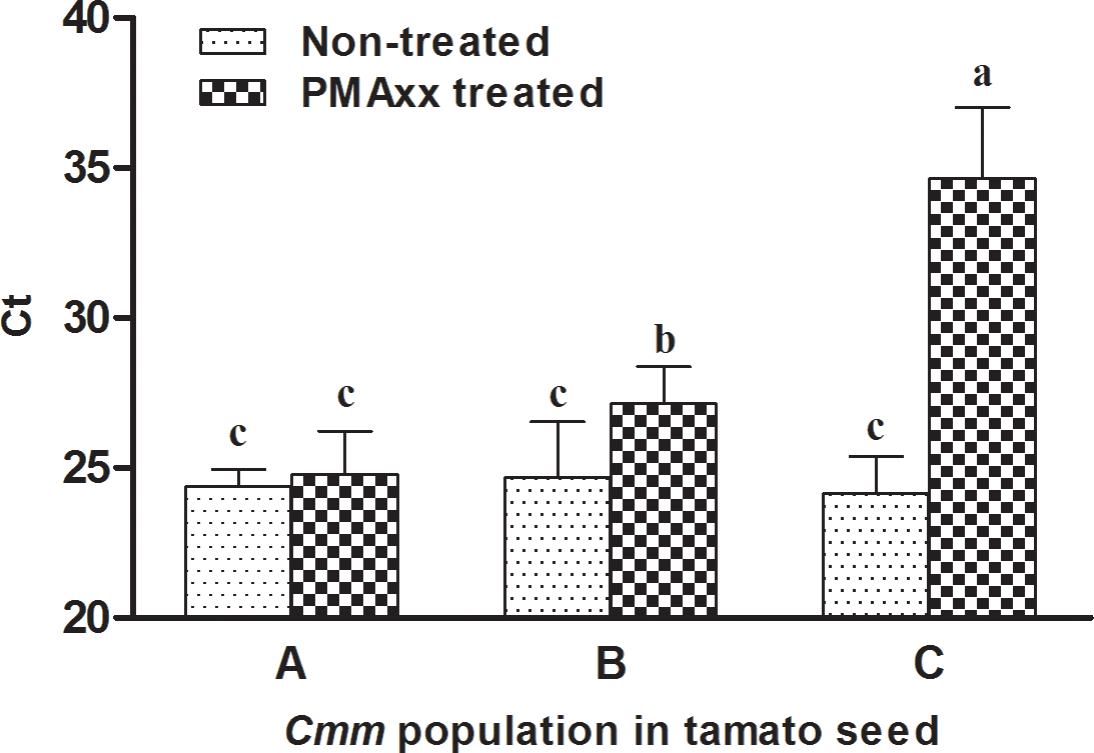
Detection of culturable, viable but non-culturable (VBNC) and dead cells of *Clavibacter michiganensis* subsp. *michiganensis* (*Cmm*) by PMAxx-qPCR from artificially inoculated tomato seed. Ten tomato seeds were soaked in log phase (A), copper-induced VBNC (B) and heat-killed (C) *Cmm* cells suspension (10^8^ CFU mL^-1^) by vacuum infiltration. After inoculation, the seed was broken by a ball mill and diluted 20-fold with 0.85% NaCl solution, followed by treatment with or without PMAxx at a final concentration of 20 μM. DNA was extracted after PMAxx treatment and used for qPCR assay. Cycle threshold (Ct) of qPCR was separated using multiple range test, and means labeled with different letters were significantly different (*P* < 0.05).

Tomato (*Solanum lycopersicum*) seed ‘908’ was confirmed as *Cmm* negative by agar plating and Bio-PCR (data not shown). The Ct values for conventional qPCR showed no difference for the seed samples inoculated with culturable, VBNC and dead *Cmm* cells, but the Ct values of PMAxx-qPCR were significantly different (Fig 4). For seed samples containing viable cells, there was no significant difference of the Ct values for PMAxx-qPCR and conventional qPCR; for seed samples containing dead cells, Ct value of PMAxx-qPCR was higher than conventional qPCR (Fig 4). The total number of *Cmm* cells in 10 tomato seeds, which inoculated with VBNC cells, was 2.53 × 10^5^ CFU mL^-1^ (Ct value of conventional qPCR = 24.68), while the number of viable *Cmm* cells was 4.45 × 10^4^ CFU mL^-1^ (Ct value of PMAxx-qPCR = 27.14). VBNC cells in the microcosm were calculated as approximately 17.57% of the total cells. Therefore, PMAxx-qPCR detected VBNC *Cmm* cells in a tomato seed sample.

## Discussion

We have demonstrated an improved and effective strategy to detect *Cmm* in the VBNC state, which cannot be otherwise detected by using conventional bio-PCR, and confirmed that the new photoactivatable dye PMAxx had higher sensitivity to differentiate viable and dead cells and less false binding to the DNA of viable cells compared to PMA. This result is supported by studies on the detection of hepatitis A virus (HAV) and Norovirus [35,39].

It has been reported that the sensitivity of PMA-qPCR varies depending on bacterial species [40]. In the present study, in the range of 10^3^ to 10^7^ CFU mL^-1^ bacteria, PMAxx worked properly, and no false amplification occurred on dead bacteria, which was an appropriate range for a precise detection. When the *Cmm* concentration increased to or was more than 10^8^ CFU mL^-1^, dead cells could be partially picked up by PMAxx-qPCR. However, it was difficult to distinguish the difference of Ct values between viable and dead *Cmm* cells at a low bacterial concentration (< 10^3^ CFU mL^-1^). Additionally, we found that a proper concentration of PMAxx improved the accuracy of detection. This result was consistent with that of Luo et al., who used a *Taq*Man probe and EMA-qPCR to detect viable *Cmm* cells [27]. Thus, *Cmm* cells at a concentration higher than 10^8^ CFU mL^-1^ or lower than 10^3^ CFU mL^-1^ should be avoided for detecting viable cells. In other words, PMA-qPCR and PMAxx-qPCR can be used when the bacterial concentration is in an appropriate range.

The consistency and reliability of PMAxx-qPCR results were confirmed by flow cytometry and culture-based methods. Because the primers are highly specific, PMAxx-qPCR could only detect *Cmm* without showing other bacteria, thus this method can be used for the precise detection of viable *Cmm* cells in tomato plants and seed. Moreover, PMAxx-qPCR requires a minimal population of *Cmm* as low as 10^3^ CFU mL^-1^, which was much lower than that (10^5^ CFU mL^-1^) by flow cytometry [14]. A similar result was obtained in assessing the viability status of *Listeria monocytogenes* in the food industry [29].

*Cmm* can survive on (externally) or in (internally) tomato seed [38], suggesting that whole seed tissues should be examined for bacterial detection. In the present study, *Cmm* was successfully detected in artificially inoculated tomato seeds, which required only 10 seeds for reliable results, although the inoculum concentration was as low as 10^5^ CFU mL^-1^ (S1 Fig). In PMAxx treatment, the turbidity of the seed extracts could influence the exposure and photolysis, as the original thick seed extracts affected the transmission of the light from halogen bulbs [31]. To ensure the PMAxx photolysis, the seed extracts must be diluted prior to light exposure. In the present study, we found that 20 times or higher dilution of seed extract generated reliable PMAxx-qPCR results (S2 Fig). However, dilution of the sample also reduces bacterial concentration, which may affect the PCR result. Apparently, the sample preparation can be further improved. Ideally, we would expect to remove plant materials from the sample without significantly reduce the bacterial concentration. Compared to log phase cells from seed extract, the Ct value of copper-induced *Cmm* cells significantly increased in the PMAxx-qPCR detection, suggesting that most of the *Cmm* cells were dead and lost their intact membrane during VBNC induction; this result was consistent with the detection of VBNC *Escherichia coli* O157:H7 cells by PMA-qPCR [26].

In conclusion, the PMAxx-qPCR method was the most effective in the detection and quantification of VBNC *Cmm* from both pure culture and tomato seeds. This method will provide a precise detection of bacterial pathogen and evaluation for risks of VBNC cells in seed lots and other plant samples.

## Supporting information

S1 Fig Correlation between cycle threshold (Ct) of quantitative real-time polymerase chain reaction and population of *Clavibacter michiganensis* subsp. *michiganensis* (*Cmm*) on samples of tomato seeds that were artificially inoculated with *Cmm*. Each group of 10 tomato seeds was treated with *Cmm* suspension at concentrations from 10^8^ to 10^4^ CFU mL^-1^ by using vacuum infiltration. The treated seeds were ground by a ball mill in 1 mL 0.85% (w/v) NaCl solution to produce a bacterial extract and DNA extraction.
(TIF)

S2 Fig Effect of seed extract dilution on the threshold cycle (Ct) of PMAxx-qPCR for detecting viable cells of *Clavibacter michiganensis* subsp. *michiganensis* from artificially *Cmm*-inoculated tomato seeds. Ten tomato seeds were treated with *Cmm* cells suspension at a concentration of 10^8^ CFU mL^-1^ for inoculation with vacuum infiltration. These seeds were ground by a ball mill in 1 mL 0.85% (w/v) NaCl solution to produce a bacterial extract. The seed extract was diluted to different concentrations (X axis) prior to treatment with PMAxx (final concentration 20 μM). The DNA was extracted after PMAxx treatment and homogeneous exposure and subsequently used for qPCR assay.
(TIF)
