# Supplementary figures and images for: Detection of Clavibacter michiganensis subsp. michiganensis in viable but nonculturable state from tomato seed using improved qPCR
Source: PLoS One. 2018 May 3;13(5):e0196525. doi: 10.1371/journal.pone.0196525 (PMC5933903; doi:10.1371/journal.pone.0196525)

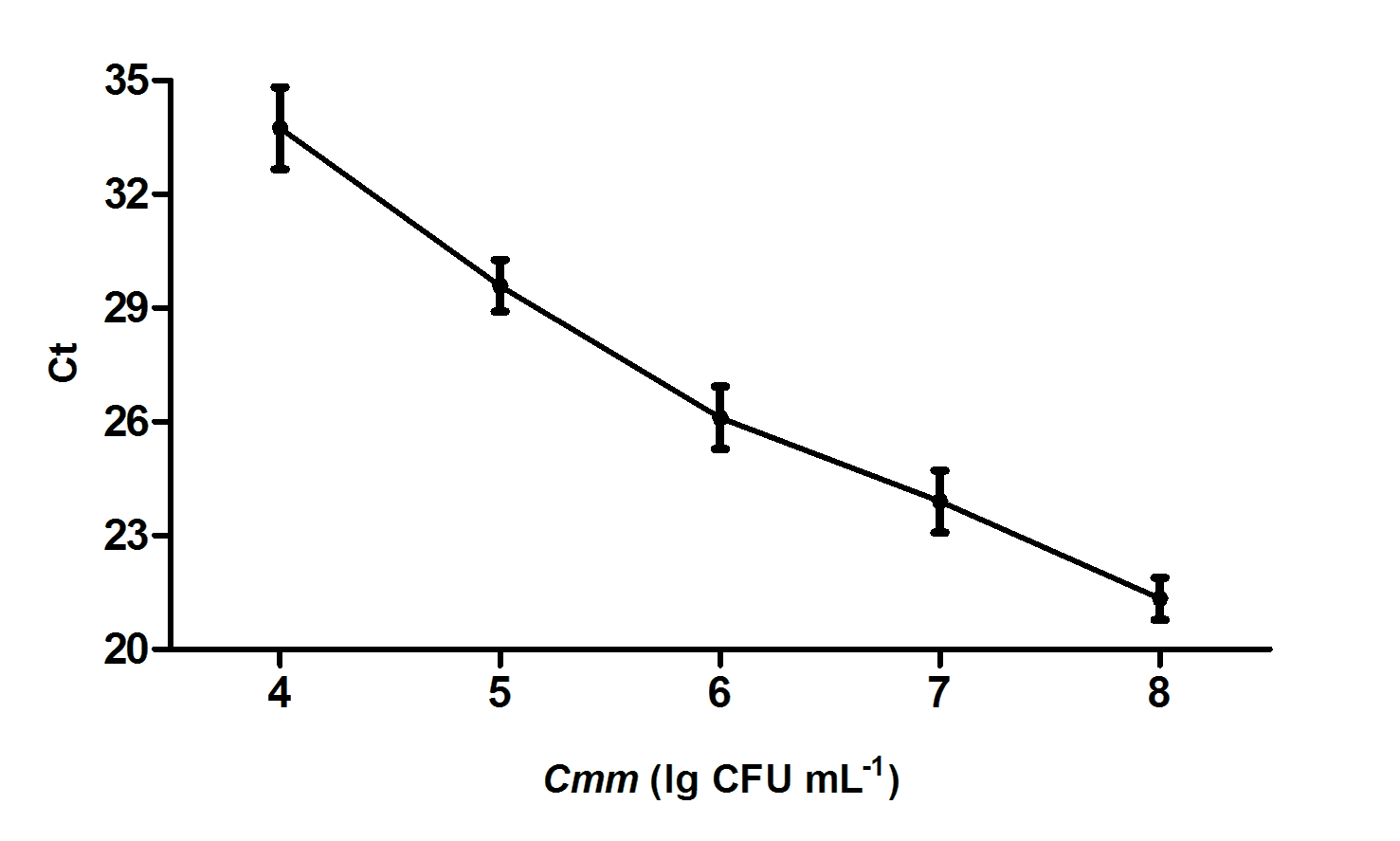

Supplement: S1 Fig — Each group of 10 tomato seeds was treated with Cmm suspension at concentrations from 108 to 104 CFU mL-1 by using vacuum infiltration. The treated seeds were ground by a ball mill in 1 mL 0.85% (w/v) NaCl solution to produce a bacterial extract and DNA extraction. (TIF) [file pone.0196525.s001.tif]

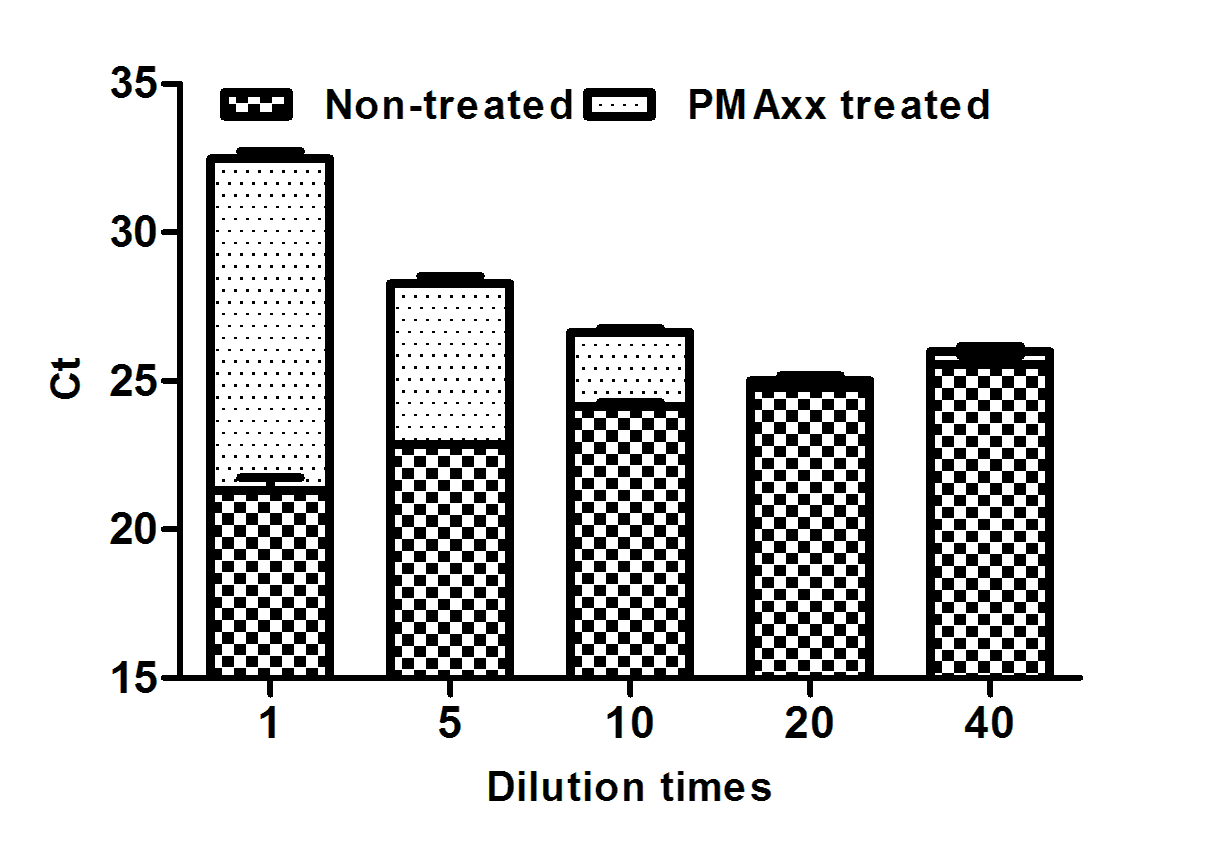

Supplement: S2 Fig — Ten tomato seeds were treated with Cmm cells suspension at a concentration of 108 CFU mL-1 for inoculation with vacuum infiltration. These seeds were ground by a ball mill in 1 mL 0.85% (w/v) NaCl solution to produce a bacterial extract. The seed extract was diluted to different concentrations (X axis) prior to treatment with PMAxx (final concentration 20 μM). The DNA was extracted after PMAxx treatment and homogeneous exposure and subsequently used for qPCR assay. (TIF) [file pone.0196525.s002.tif]
